# Reconceptualising emotion dysregulation in the context of middle childhood: A scoping review of reviews

**DOI:** 10.1002/jcv2.12296

**Published:** 2024-11-29

**Authors:** Evelyn Mary‐Ann Antony, Nadin Beckmann, Steve Higgins

**Affiliations:** ^1^ School of Education Durham University Durham UK

**Keywords:** conceptualisation, emotion dysregulation, middle childhood, scoping review, youth psychopathology

## Abstract

**Background:**

Recent research has suggested that emotion dysregulation (ED) is a key mechanism which explains the associations between mental health illnesses, including attention deficit hyperactivity disorder and internalising problems, among youth. However, literature reviews have led to mixed and inconclusive findings on the conceptualisations of ED. Specifically, understanding ED in the context of middle childhood, otherwise known as ‘the forgotten years’, may be crucial, as it serves as a significant developmental stage, where children develop a sense of self and rationalise their emotions. This scoping review aims to address the present challenge of conceptualising ED during middle childhood.

**Methods:**

ProQuest, PsycINFO, Scopus and Web of Science were searched in October 2024. Articles were included if ED and emotional dysregulation (including positive emotions) were conceptualised, if the paper was a review (i.e., scoping, literature, meta‐analysis etc), participants of the target age range (6–12‐year) were in the sample, and papers were in English. Studies were excluded if the sample included other age groups, such as infants and pre‐schoolers, alongside focussing on other phenomena, such as self‐regulation.

**Results:**

The current scoping review explored the conceptualisations drawn from research studies conducted in the United States, Germany, Austria, France, and Australia. 11 studies were included in this review and two key themes were extrapolated (a) issues with processing emotions (i.e., emotion generation/reactivity) and (b) issues with adopting appropriate emotion regulation strategies and the failure in doing so, leading to issues in attaining goal‐directed behaviours.

**Conclusions:**

The findings support the importance of considering how ED should be measured, based on holistic perspectives of the mechanism, alongside clinical screening for emotional deficits at earlier stages of life.

## Graphical abstract

Middle childhood (ages 6–12) represents a key developmental stage wherein young people develop appropriate emotion regulation skills, including distraction and distancing strategies, for autonomously regulating stress. Nevertheless, during this stage of rapid emotional development and vulnerability, sudden fluctuations in emotions including temper tantrums, outbursts and self‐soothing, have yet to be disentangled, particularly in relation to emotion dysregulation and the emergence of youth psychopathological issues. The purpose of this study was to reconceptualise emotion dysregulation in the context of middle childhood. Our findings extrapolated two key themes from the 11 included studies (a) issues with processing emotions (i.e., emotion generation/reactivity) and (b) issues with adopting appropriate emotion regulation strategies and the failure in doing so, leading to issues in attaining goal‐directed behaviours.

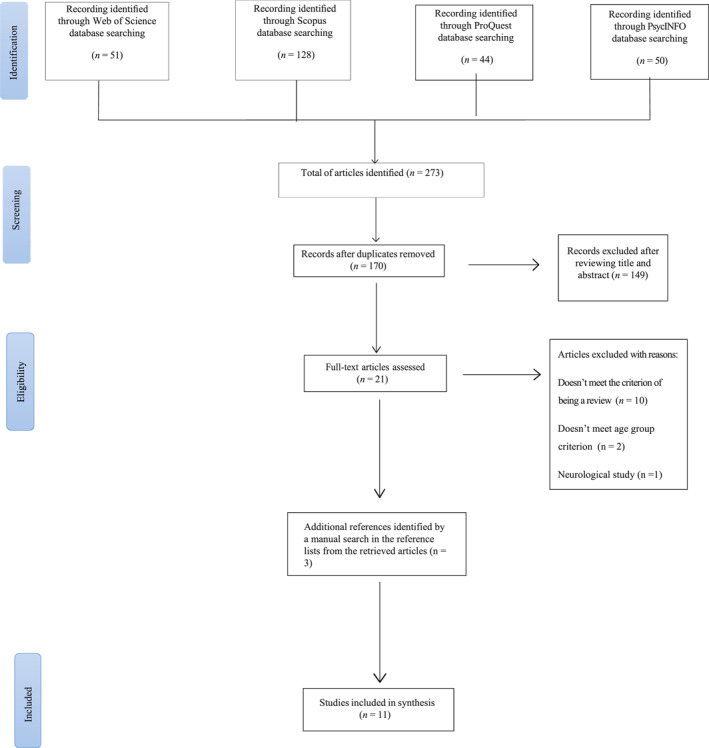

**Key points**

- Emotion dysregulation (ED) is a key mechanism which explains the associations between and across mental health issues, including attention deficit hyperactivity disorder and internalising problems.
- Middle childhood is a key developmental stage where children develop a sense of self and rationalise their emotions.
- Our novel study aims to address the present challenge of conceptualising ED during middle childhood, otherwise known as ‘the forgotten years’.
- ED may provide key clinical implications, including the potential to screen for emotional deficits during early childhood, thus preventing and minimising the accumulation of mental health issues over the life course.

## BACKGROUND

Mental health problems are more prevalent during childhood and adolescence, than physical health problems (Goodman et al., 2011). This prevalence can lead to debilitating issues including attaining lower grades at school, engaging in risky behaviours (i.e., substance misuse), and increased risks of engaging in criminality during adulthood (Fletcher & Wolfe, 2008). During the key developmental stage of middle childhood (ages 6–12), by children gain better cognitive control, make independent decisions, and have an active role within families and communities (Mah & Ford‐Jones, 2012). Yet, 18% of children aged 7–16 years old display symptoms of developing probable mental health disorders, (Newlove‐Delgado et al., 2022). To address such issues among youth, researchers have turned to *psychological mechanisms,* defined as processes or events that lead to changes in psychological outcomes (Sripada et al., 2016). To this end, emotions are a multifaceted psychological mechanism by which individuals respond to social cues and environmental threats (Smidt & Suvak, 2015).

According to Gross (1998), emotion regulation refers to shaping which emotions one has, when one has them, and how one expresses these emotions, with key features including (a) the activation of a regulatory goal, (b) the engagement of regulatory processes), and (c) the modulation of the emotion trajectory. Across a vast range of mental illnesses, emotional disorders (including anxiety and depression) are of particular interest to researchers, as individuals experience impairments in skills including expressing emotions appropriately in social situations (Carl et al., 2013). This deficiency in emotion regulation, known as emotion or emotional *dysregulation*, increases the risk of psychopathological disorders (e.g., attention deficit hyperactivity disorder [ADHD], bipolar disorder) developing among youth, alongside potential co‐occurring issues in social functioning and poor family relations (Low et al., 2019; Vogel et al., 2021). Despite ED being recognised as a core feature of psychopathological disorders, there is a lack of consensus among researchers around how ED can be conceptualised, particularly in middle childhood.

### An overview of emotion dysregulation

Several studies have suggested that ED can be defined as unpredictable shifts from having a normal mood to feelings of lowness or mild excitement, often accompanied by frequent brief outbursts and feelings of being overwhelmed (Anastopoulos et al., 2011; Barkley, 2014; Christiansen et al., 2019; Corbisiero et al., 2013).Yet, literature highlights differences, when accounting for factors associated with ED. For instance, Rogosch and Cicchetti (2005) suggested that ED is defined by affective negativity, irritability, lability, suicidal and self‐harm behaviour, impulsivity, and extreme conflict, leading to struggles in interpersonal relationships with peers and adults. Furthermore, Cole and Hall (2008) presented a different type of conceptualisation, emphasising ineffective attempts at regulation, behavioural interferences due to emotions, and inappropriate expressions of emotions (e.g., humour). Importantly, ED is used to describe contexts where existing deficits exist (including ADHD), compared to other interchangeable terms (e.g., emotion lability), which do not consider pre‐existing conditions (Christiansen et al., 2019).

A variety of interchangeable terms have been used to describe ED, including ‘emotion lability’, ‘emotion impulsivity’ and ‘emotion instability’, leading to issues in conceptualising the phenomenon (Faraone et al., 2019; Low et al., 2019). For example, emotion lability is defined as *unpredictable* shifts towards negative emotions such as sadness, dysphoria, and anger, with an intensity that is deemed culturally inappropriate to the situational context (Barkley, 2014). Furthermore, ‘affect’: a superordinate category accounting for both emotions and moods, has been associated with dysregulation (Niven, 2013). There are overlapping features between ‘affect dysregulation’ and ‘ED’, including difficulties with calming down after being upset and heightened reactivity to strong emotions (Narendorf et al., 2016).

Overall, due to the lack of consensus on conceptualising ED among researchers (whether this includes negative emotions or inappropriate expressions of emotions), there is a need to determine what dimensions are more prevalent in the presentation of dysregulation (i.e., emotion reactivity, emotion generation).

### Middle childhood

Middle childhood (children aged 6–12) represents a key developmental stage in determining later mental health, where the consolidation and integration of emotions, skills, and personal knowledge, enable children to develop better self‐awareness and in‐depth interpersonal connections, with their families, peers, and teachers (Petersen et al., 2010; Sharpe et al., 2016). Yet, middle childhood has been deemed the ‘forgotten years’ of development, as prior literature has mostly focussed on developmental trajectories during late childhood and early adolescence (Mah & Ford‐Jones, 2012). Importantly, research stipulates that middle childhood represents a stage of introspection, when children develop self‐awareness, realising that they are distinct from others, as well as developing skills in morality and decision‐making (Damon & Hart, 1982; Mah & Ford‐Jones, 2012).

Middle childhood also represents the developmental stage wherein young people develop appropriate emotion regulation skills. During this process, children use multiple strategies, including distraction and distancing strategies, for autonomously regulating their emotions and managing stress (Skuse, 2011). Evidently, when children experience positive emotional development in middle childhood, they are equipped with the ability to understand their sense of self and can cope with the emotional demands in evolving friendships. Nevertheless, during this stage of rapid emotional development and vulnerability, it is possible to differentiate between typical emotional functioning and the emotional functioning of those with mental health issues (Cole et al., 2008). For instance, typically developing children's emotional outbursts may comprise an acute peak of anger or distress, which quickly declines following self‐soothing or comfort‐seeking from an adult, in comparison to children with mental health issues (i.e., ADHD), who might demonstrate persistent, excessive, and destructive peaks of intense emotions during such tantrums (Panayiotou & Humphrey, 2018). Furthermore, emotion regulation continues to predict cognitive and social functioning as children enter middle childhood, (Berkovits & Baker, 2014; Rydell et al., 2007). Crick & Dodge's (1994) model of children's social adjustment also highlights that emotional experiences can influence children's responses to social situations. Linking this to emotion regulation research, the social adjustment model may aid researchers' understanding of how children respond in a socially compatible manner, with regards to goal‐directed behaviour, particularly when their emotions might otherwise interfere with such interactions. Goodman and Southam‐Gerow (2010) found that children struggling to regulate negative emotions including teasing may adopt aggressive or ruminative coping strategies, impacting prosocial behaviour and social acceptance.

Taken together, the evidence presented here suggests that sudden fluctuations in emotions during temper tantrums, outbursts and self‐soothing, need to be disentangled to better understand how the process of dysregulation emerges and how it is maintained (in cases of psychopathology vs. everyday situations). The relative paucity of research in this area presents a gap that needs to be explored—enabling a better understanding of how dysregulation is conceptualised in this under researched age group.

### The present study

The goal of the present scoping review is to report and discuss existing *academic conceptualisations* of ED and emotional dysregulation in the context of an under researched developmental stage, middle childhood (ages 6–12). Literature suggests that during middle childhood children realise that they are distinct from others and begin to rationalise their own emotions, developing their self‐esteem (Mah & Ford‐Jones, 2012). This scoping review will be a ‘review of reviews’, whereby existing literature reviews that conceptualise ED will be critically evaluated, with reference to middle childhood and key characteristics of dysregulation, including excessive emotions. After summarising the current state of knowledge, we discuss the limitations of existing research and future research directions, alongside implications for clinical practice.

## METHODS

### Scoping review

Recent literature has sought to provide guidance to researchers on determining whether to adopt a systematic or scoping review approach. The purpose of a scoping review is to examine the breadth of research activity, where it may be unclear what other, more specific questions can be posed (Anderson et al., 2008; Arksey & O’Malley, 2005). Moreover, scoping reviews aim to determine if the results from the review should be used to answer a clinically meaningful question or to inform practice through the evidence collected (Munn et al., 2018). This scoping review was conducted, using the following five‐stage methodological framework as outlined by Arksey and O’Malley (2005): (a) identifying the research question, (b) identifying relevant studies, (c) study selection, (d) charting the data, and (e) collating, summarising, and reporting the results.

***Research Question.*** This scoping review is guided by the following research question: *How can ED be conceptualised in the context of middle childhood?*

***Identifying Relevant Studies and Study Selection.*** As discussed previously, a range of terms have been used to describe ED, including ‘affect dysregulation’ and ‘emotion impulsivity’, denoting differences in the severity and presentation of symptoms. Emotion dysregulation accounts for pre‐existing psychopathological conditions (e.g., ADHD), compared to other terminologies (see Background). Searching was conducted on 9th October 2024, using the databases: Web of Science, Scopus, ProQuest and PsycINFO with the following key words (using Boolean operator “AND”): emotional AND emotion AND dysregulation AND childhood AND review. Using both ‘emotional’ and ‘emotion’ dysregulation were key in determining the breadth of existing literature. Although the key word of ‘childhood’ was applied to the search strategy relatively broadly to determine the magnitude of research, linked to ED, ‘childhood’ specifically referred to middle childhood (ages 6–12), while screening for full texts. Papers with a sole focus on the target population ‘adolescents’ were therefore excluded. Although not required for scoping reviews, peer‐reviewed articles were specified during the search to restrict the scope of the review to higher quality research. Moreover, only papers in the English language were included in all searches across the specified databases, due to time and cost restraints in utilising translation services. We report our findings according to the Preferred Reporting Items for Systematic reviews and Meta‐Analyses extension for Scoping Reviews (PRISMA‐ScR) Checklist (Campbell et al., 2020; Tricco et al., 2018).

***Charting the Data.*** With regards to charting the data, a stringent exclusion criterion was applied at the screening phase for abstracts and titles, but where unclear work was included, it was examined further at the full text stage. Importantly, studies that were neurological (Beauchaine, 2015) or reviews that focussed on measurement issues in ED were not included (Althoff & Ametti, 2021), as these did not meet our criteria. Table 1 provides a detailed overview of the inclusion and exclusion criteria under each subject heading: population/sample, phenomenon of interest, study design, language, and date range. Table 2 presents an overview of the search strategy for each database.

**TABLE 1:** Inclusion and exclusion criteria.

Criterion	Inclusion	Exclusion
Population/Sample	Childhood Age range: Middle childhood (ages 6–12) Whole population (i.e., all children including those in specific subgroups of psychiatric disorders (ADHD), anxious children, effects of maltreatment/abuse etc.).	Infants Toddlers Pre‐school Adolescents Adults
Phenomenon of interest	Emotional and emotion dysregulation (includes dysregulation of positive emotions)	Self‐regulation (cognitive); meta‐cognition
Design	Review (narrative, scoping, systematic, meta‐analysis, tertiary, critical, comprehensive, conceptual).	Empirical studies Reviews of neurological/pharmacological studies
Language	English	Languages other than English
Date range	All years	None

Abbreviation: ADHD, attention deficit hyperactivity disorder.

**TABLE 2:** Search strategy.

Scopus TITLE‐ABS‐KEY (emotional AND emotion AND dysregulation AND childhood AND review) AND (LIMIT‐TO (LANGUAGE, “English”))
Web of Science [*abstract]* ‘emotional’ AND ‘emotion’ AND ‘dysregulation’ AND ‘childhood’ AND ‘review’; limit to language = English
PsycINFO [*abstract]* ‘emotional’ AND ‘emotion’ AND ‘dysregulation’ AND ‘childhood’ AND ‘review’; limit to language = English
PubMed [*abstract]* ‘emotional’ AND ‘emotion’ AND ‘dysregulation’ AND ‘childhood’ AND ‘review’; limit to language = English

**FIGURE 1:**
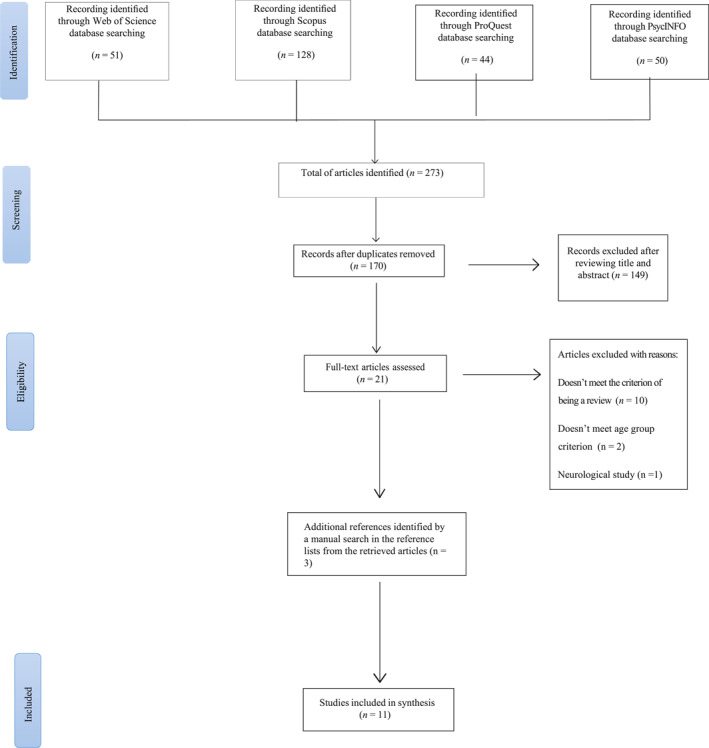
Flowchart of study.

***Scoping Review.*** The original search findings from Web of Science, Scopus, ProQuest and PsycINFO on 9th October 2024 yielded a total of 273 papers (51, 128, 44 and 50 papers, respectively). After removing duplications, 170 articles remained. Following this, 149 publications were excluded after screening the title and abstract, with 21 papers remaining. 13 studies were excluded after reviewing the full text, according to the inclusion and exclusion criteria, with 8 relevant papers being identified. Inter‐rater reliability (IRR) was examined to check the consistency of decisions based on the inclusion and exclusion criteria at the initial screening phase, alongside the eligibility phase. Papers were selected using a random generator for further verification by NB and SH.; 10% of the initial sample was chosen at random for NB (27 papers out of 273) and 43% (9 papers out of 21) was chosen at random for SH. Typically, 10% is the minimum sample for IRR, thus the proportions utilised for conducting IRR by NB and SH was appropriate. Results for the initial screening phase show high agreement between EMA and NB, with only 1 case of disagreement, which was investigated further by the authors and rectified, following discussions. For the eligibility phase, there was complete agreement between EMA and SH. Additionally, backward citation searching, which involves manually checking reference lists from retrieved articles, was conducted for this review. This additional method identifies potentially relevant studies that might not be retrieved by other search methods (Briscoe et al., 2020; Haddaway et al., 2022). From the backward citation searching, 3 articles were identified as being potentially relevant. In total, 11 articles were included in the present review. Figure 1 shows the research flowchart based on the PRISMA‐ScR guidelines. The PRISMA flowchart and search strategies were pre‐registered in October 2023 on Open Science Framework (Antony et al., 2024).

## RESULTS

The Garrard Matrix Method (2017) was used to critically evaluate articles, extract and tabulate study components, compare findings, and synthesise recommendations, as well consolidating conceptualisations of ED into a single table and extracting key themes. During the synthesis stage the articles were read by the first author multiple times to inductively derive similarities and differences of ED conceptualisations. Table 3 presents the resulting matrix.

**TABLE 3:** Garrard matrix of emotion dysregulation (ED) conceptualisations.

Author/Year/Country	Paper title	Journal	Article type	Study Aim(s)	Conceptualisation of emotion dysregulation
Bunford et al. (2015) *USA*	ADHD and emotion dysregulation among children and adolescents	*Clinical Child and Family Psychology Review*	Comprehensive review	(1) To examine the association between emotion dysregulation and ADHD among youth. (2) To examine whether emotion dysregulation is associated with risky behaviours and social impairments with ADHD.	*“Emotion dysregulation is an individual's inability to exercise any or all aspects of the modulatory processes involved in emotion regulation, to such a degree that the inability results in the individual functioning meaningfully below his or her baseline.”*
Chua et al. (2021) *USA*	Treatment of childhood emotion dysregulation in inpatient and residential settings	*Child and Adolescent Psychiatric Clinics of North America*	Systematic literature review	(1) To summarise existing strategies for the management of emotion dysregulation in inpatient and residential settings.	*“Emotion dysregulation, defined as impairment in the psychological processes that maintain a child's emotional and behavioural self‐control, may be a result of lagging development skills (i.e., trait) or a temporary loss of previously acquired skills (i.e., state).”*
Golombek et al. (2020) *Germany*	The role of emotion regulation in socially anxious children and adolescents: a Systematic review	*European Child & Adolescent Psychiatry*	Systematic literature review	(1) To utilise a process model of emotion regulation as a framework for understanding emotion regulation in children and adolescents with social anxiety.	*“Emotion dysregulation can be conceptualised as a state in which one's attempts to regulate emotions fail to achieve emotion‐related goals despite best efforts, which is associated with psychopathology.”*
Graziano and Garcia (2016) *USA*	Attention‐deficit hyperactivity disorder and children's emotion dysregulation: A meta‐analysis	*Clinical Psychology Review*	Meta‐analysis	(1) To examine whether there are associations between ADHD and various emotion dysregulation domains, after accounting for co‐occurring conduct problems.	*“Broadly speaking, emotion dysregulation occurs when an individual fails to modify an emotional state so as to promote adaptive behaviours that are necessary to accomplish his/her goals.”*
Gruhn and Compas (2020) *USA*	Effects of maltreatment on coping and emotion regulation in childhood and adolescence: A meta‐analytic review	*Child Abuse & Neglect*	Meta‐analysis	(1) To investigate the impact of early‐life maltreatment on coping and emotion regulation processes during childhood and adolescence (5–18 years).	*“Emotion dysregulation can be defined as failing to account for strategies behind dysregulated responses, leading to attempts to interfere with and to delineate controlled and automatic responses.”*
Harvey et al. (2019) *Australia*	Dialectical behaviour therapy for emotion regulation difficulties: A systematic review	*Behaviour Change*	Systematic literature review	(1) To investigate and evaluate the current evidence to understand the effectiveness of dialectical behaviour therapy in improving emotion regulation difficulties.	*“Emotion dysregulation evolves* via *an interaction between the individual's biology and their social environment, beginning in childhood.”*
Paulus et al. (2021) *Germany & Austria*	Emotional dysregulation in children and adolescents with psychiatric disorders. A narrative review	*Frontiers in Psychiatry*	Narrative literature review	(1) To provide an overview on the various aspects of emotion dysregulation in children and adolescents with psychiatric disorders, focussing on clinical characteristics, prevention, and therapy.	*“Emotion dysregulation (ED) is a transdiagnostic construct defined as the inability to regulate the intensity and quality of emotions (such as, fear, anger, sadness), in order to generate an appropriate emotional response, to handle excitability, mood instability, and emotional over reactivity, and to come down to an emotional baseline.”*
Schipper and Petermann (2013) *Germany*	Relating empathy and emotion regulation: Do deficits in empathy trigger emotion dysregulation?	*Social Neuroscience*	Critical review	(1) To present different studies investigating the relation between empathy and emotion regulation and discuss whether empathy triggers emotion regulation.	*“Deficits in one or both, affective arousal and emotion understanding, are key aspects of emotion dysregulation.”*
Shaw et al. (2014) *USA*	Emotion dysregulation in ADHD	*American Journal of Psychiatry*	Mixed studies review (systematic literature review & meta‐analysis)	(1) To explain the overlap between emotion dysregulation and ADHD.	*“Emotion dysregulation encompasses 1) emotional expressions and experiences that are excessive in relation to social norms and are context inappropriate; 2) rapid, poorly controlled shifts in emotion (lability); and 3) the anomalous allocation of attention to emotional stimuli.”*
Vacher et al. (2020) *France*	Efficacy of psychosocial interventions for children with ADHD and emotion dysregulation: a Systematic review	*Psychiatry Research*	Systematic literature review	(1) To examine the effects of psychosocial interventions in children and adolescent with ADHD and emotion dysregulation by focussing on their efficacy for emotion dysregulation management.	*Definitions of emotion dysregulation are those from Bunford* et al. *(* 2015 *) and Shaw* et al. *(* 2014 *)*.
Vogel et al. (2023) *USA*	Review: Defining positive emotion dysregulation: Integrating temperamental and clinical perspectives	*Journal of the American Academy of Child & Adolescent Psychiatry*	Critical review	(1) To introduce clinicians to research associated with dysregulated positive affect and to review recent studies of positive affect dysregulation in clinical populations.	*“Emotion dysregulation refers to a maladaptive process of emotional experiences or expressions experienced either too intensely or too enduringly.”*

Abbreviation: ADHD, attention deficit hyperactivity disorder.

Of the 11 articles included, four papers were systematic literature reviews, followed by two of each: meta‐analysis, critical review, and comprehensive review; and finally, one narrative review and one combined study (consisting of a meta‐analysis and a systematic literature review). The research focus across the studies investigated ED in the context of: (a) mental health illnesses and (b) psychosocial interventions. Drawing upon commonalities from the 11 articles, the following key characteristics associated with conceptualising ED were inductively derived: (a) issues with processing emotions, including emotion generation and emotion reactivity, (b) issues with adopting the appropriate response or strategy to attain goal‐directed behaviour. Besides this, one paper presented a conceptualisation that didn't fit into either category, reflecting upon the role of the individual and being situated in a social environment.

### Theme 1: Emotion generation and reactivity

Six studies provided insights on the role of maladaptive emotion generation and reactivity, in conceptualising ED. Importantly, two subthemes emerged from the overall theme of emotion generation and reactivity: (a) issues with functioning, from a cognitive regulation standpoint and (b) issues with the intensity and severity of emotion experiences. For instance, Bunford et al. (2015) illustrated that ED occurs due to impairments in functioning, whereby the individual lacks the ability to regulate their own emotions at the optimum baseline level, leading to psychopathological issues. Conversely, the other five studies from Paulus et al. (2021), Schipper and Petermann (2013), Shaw et al. (2014), Vacher et al. (2020) and Vogel et al. (2023) focus on the severity and intensity of emotions experienced during dysregulation. Furthermore, Shaw et al. (2014) presents an extended description of the process, whereby excessively experienced emotions may lead to behaviours that are deemed as socially and culturally unacceptable. Importantly, Paulus et al. (2021) presents ED as a transdiagnostic construct, implying that it involves several dimensions, with reference to negative emotions including sadness, fear, and anger. Overall, the conceptualisation presented by Bunford et al. (2015) do not fully consider functional impairments (e.g., shortened attention span): it is unclear when or how such impairments arise, suggesting that there is a need to disentangle the complex relationship between dysregulation and associated cognitive deficits. Furthermore, conceptualisations that focus on the presentation of dysregulation symptoms have weighed more on negative emotions, despite being previously defined as a multidimensional construct. Therefore, other factors including the failure to regulate positive emotions, may also be important to investigate, to gain a holistic overview of the mechanism.

### Theme 2: Adopting appropriate strategies

Four studies ‐ Chua et al. (2021); Graziano and Garcia (2016); Gruhn and Compas (2020) and Golombek et al. (2020) ‐ provided insights into ED conceptualisations, by focussing on the failure to achieve goal‐directed behaviours. For example, Graziano and Garcia (2016), alongside Golombek et al. (2020) reflect upon the importance of failing to modify an emotional state, leading to issues in achieving emotion‐related goals (see Discussion). Crucially, both papers discuss ED in the context of emotional states, indicating that negative emotions, including sadness, may be an outcome of attempting to adopt an appropriate strategy, but failing to attain goal‐directed behaviours, such as self‐soothing. Moreover, Chua et al. (2021) and Gruhn and Compas (2020) highlight other aspects of adopting appropriate strategies, referring to self‐control and delineating from controlled and automatic responses. Chua et al. (2021) suggests that impairments in both emotional and behavioural self‐control may be due to developmental issues, including problems with acquiring relevant skills or developmental delays, which lead to dysregulation. Taken together, developmental trajectories and failing to adopt appropriate coping mechanisms may play a key role in shaping the course of ED and must be further explored to understand its implications for associated impairments among youth.

Most ED conceptualisations presented do not account for development (see Table 3) and instead, reflect upon issues in processing emotions, reaching goal‐directed behaviours and overall impairments in functioning. Harvey et al. (2019) offered a developmental perspective on ED, describing it as an evolving process between individuals and their environment starting in childhood. This raises questions about external influences shaping ED trajectories, alongside bidirectional relationships, which have not been fully explored in prior research.

## DISCUSSION

The scoping review sought to answer a clinically meaningful question: *how can ED be conceptualised in the context of middle childhood?* Overall, findings suggest that there are two key factors associated with conceptualising ED: (a) primarily issues with processing emotions during some stage, (e.g., emotion generation/reactivity), alongside (b) issues with adopting the appropriate coping strategy (e.g., acceptance, behavioural avoidance, experiential avoidance; Skinner & Zimmer‐Gembeck, 2007). Crucially, most conceptualisations do not account for developmental stages which may be an important consideration, in the context of emotional development during middle childhood and adopting appropriate coping mechanisms in social situations (e.g., peer victimisation). Furthermore, external influences including parenting, school life and accessibility to extracurricular activities may also play a key role in shaping ED trajectories.

### Coping mechanisms and emotion dysregulation

Prior literature emphasises understanding the development of ED, including how strategies evolve, when they become effective, and how ineffective strategies change with age (Cole et al., 2019). For instance, a child may use avoidance or dissociation to cope with anxiety in familial conflict, but these strategies may become ineffective in adolescence due to biological changes including puberty (Schäfer et al., 2017). Indeed, research focussing on developmental differences during childhood and coping mechanisms have been difficult to integrate. Skinner and Zimmer‐Gembeck (2007) propose that coping definitions borrowed from adulthood may not apply directly to childhood, lacking developmental frameworks for this stage. During middle childhood, internalising coping (e.g., blaming oneself for being too emotional) acts as a mediator between shyness and internalising difficulties (e.g., loneliness and negative affect; Findlay et al., 2009). Linking this to the current review, Gratz and Roemer (2004) identify six deficits in emotion regulation that may hinder coping: (a) lack of emotional awareness, (b) lack of emotional clarity, (c) nonacceptance of emotional responses, (d) impulse control difficulties, (e) limited access to regulation strategies, and (f) difficulty engaging in goal‐directed behaviour when aroused. Gross and Barrett (2011) suggest that factors regulating emotion onset may prevent unwanted emotion activation or enhance desired ones through situation selection or modification. Taken together, these findings represent the significance of considering coping mechanisms into conceptualisations of ED, as it could lead to a better understanding of how children manage emotionally challenging situations in real‐life contexts.

### Considering external factors for emotion dysregulation

The present review has considered key characteristics in ED conceptualisations, including functioning, the severity of emotions, and failing to reach goal‐directed behaviours. Yet, external factors including adverse childhood experiences (ACEs) and bidirectional relationships between the child and the environment, may also serve a key purpose in understanding dysregulation trajectories. From a developmental standpoint, ACEs, an umbrella term encompassing trauma exposure, parental psychopathological issues, and family dysfunction, put young people at risk for functional long‐term impairments (Woods‐Jaeger et al., 2018). Familial conditions may be an important characteristic to consider in ED conceptualisations, as literature suggests that family members' emotional expressions and their predictability shape how children manage their emotions (Thompson, 2019).

Social constructivist theorists emphasise the significance of individuals developing themselves through interactions with others, as well as the environment in which they reside (Kim, 2001). As presented in the Results, the conceptualisation from Harvey et al. (2019) considers the relationship between the individual (the child) and its social environment, with regards to dysregulation emerging. Oloye and Flouri (2021) explored the role of the home environment on children's self‐regulation, illustrating that quiet home environments positively influence a child's ability to regulate, compared to disorganised environments. Furthermore, children with poor self‐regulation and higher levels of emotional intensity have less constructive reactions to anger when interacting with peers, such as avoidance and escape (Eisenberg et al., 1994). Linking this to the present review, children with greater ED and lower independence (e.g. throwing tantrums) are more likely to cause disorder in the home and reduce their chances of success in social interactions (Barnes et al., 2013). Overall, the role of the home environment, alongside schooling and extracurricular activities, may be important perspectives to consider in future conceptualisations.

### Strengths

The present study has notable strengths. Firstly, while considering the ‘whole population’ as a sample, including typically developing children and those with pre‐existing mental health issues, the authors were able to attain a wider range of academic conceptualisations, during the screening process. We acknowledge that the papers retrieved from the synthesis stage discussed ED solely in the context of psychopathology among youth. However, this evidence is concurrent with prior literature, which has illustrated associations between ED and mental health problems (see Background).

Another key strength in this paper was the use of Garrard Matrix Method (2017), enabling better identification of key differences and similarities between conceptualisations. In doing so, the emergence and extraction of key themes, including issues with processing emotions and issues with attaining goal‐directed behaviours, were better identified with efficiency and ease.

Importantly, to the best of the authors' knowledge, this study was the first to investigate how ED can be conceptualised in middle childhood. By considering key factors including the environment and developmental perspectives, which influence ED symptoms, thus shaping our understanding of the mechanism. Such perspectives will be pertinent in follow‐up studies, which may choose to focus on how ED is measured in this age group.

### Limitations

There are several limitations to address. Firstly, we decided to solely focus on ED, as it considers pre‐existing mental health conditions, as opposed to other interchangeable terminologies. While recognising this in our review, additional synonyms such as ‘emotion lability’ and ‘emotion impulsivity’, may have retrieved a better breadth of literature, subsequently leading to different results in the present paper.

Secondly, we conducted a ‘review of reviews’, as opposed to empirical studies or clinical trials. However, it is important to consider that the reviews were different in methodological approach (i.e., narrative, systematic), thus raising questions about making valid comparisons between the papers. While our focus was on conceptualisations, the context in which these were identified and evaluated were all different. We note that this may not have had a direct influence on our findings but may be a key factor to focus on in similar studies.

Finally, conceptualisations of ED were limited to the field of psychology, and we decided to explicitly exclude conceptualisations from specific fields, including neurology and pharmacology. By not considering interdisciplinary perspectives, we may have missed other conceptualisations of ED, including cognitive and social changes (Crick & Dodge, 1994), underscoring the need for a holistic overview of the mechanism.

### Implications

In the present scoping review, reporting and discussing existing conceptualisations of ED may inform a better understanding of measuring the mechanism (including measurement invariance issues and temporal dynamics; Munn et al., 2018). Moreover, identifying key symptoms of ED during middle childhood may contribute towards better clinical assessments of emotional deficits, as well as enabling better prevention of severe mental health illnesses at later stages of life, alongside the risk of developing crime‐related behaviours and profound issues with physical health (Ramchandani et al., 2017). In turn, prevention of such profound illnesses may create opportunities for improving ED deficits during childhood. Antony (2022) has explored associations between Bronfenbrenner's ecological systems theory (1979), where children are at the ‘centre’, developing key relationships with parents, teachers, and communities, and fostering resilience during childhood, highlighting that school‐based interventions may enable children to combat academic challenges and emotional hurdles.

### Future directions

Future studies should consider emotional *valence*—the degree to which an emotion is positive or negative—in conceptualisations of ED. Several studies have investigated dysregulation in the context of negative emotions (e.g., fear and sadness), yet have not fully explored the extent to which dysregulation may also account for the failure to regulate positive emotions (Vogel et al., 2023). Furthermore, emotional *granularity*—the ability to make fine‐grained distinctions in one's affective feelings—may also be an important consideration for future conceptualisations of ED. Wilson‐Mendenhall and Dunne (2021) stipulated that individuals with higher granularity have better abilities to distinguish between negative emotions, from anger to loneliness, compared to those with lower emotional granularity. Additionally, Somerville et al. (2023) investigated emotion *controllability beliefs*, which suggests that individuals who believe emotions are relatively controllable, are more likely to attempt to regulate their emotions and to persist in these efforts. This may be relevant for conceptualisations of dysregulation, as those that have little control over their emotions will experience difficulties in emotion regulation and lack the effort or motivation to regulate. Taken together, emotional granularity and emotion controllability beliefs are potential facets of dysregulation that need to be considered in prospective research.

## CONCLUSION

The current scoping review identified key conceptualisations of ED associated with middle childhood. The conceptualisations did recognise facets of dysregulation, such as functional impairments, negative emotions and issues with attaining goal‐directed behaviours. Therefore, additional factors, including (a) coping mechanisms during middle childhood, (b) the role of the environment, and (c) emotional granularity, and emotion controllability beliefs, should be considered in future conceptualisations of the mechanism. Furthermore, most of the conceptualisations did not account for developmental milestones, despite the reviews setting the context by focussing on ‘youth’, illustrating the need to disentangle potential trajectory differences among children. Identifying different developmental stages and acknowledging dynamic interactions within ED provides evidence for recognising and addressing the multiple facets of the mechanism. Overall, ED has often been defined by the demands of the immediate social situation and the goals of the individual, rather than as a global construct. Crucially, what is considered ‘optimal’, in the context of dysregulation, can vary depending on context, individuals, and pre‐disposed conditions. Future research should investigate the aforementioned factors for a better assessment of emerging youth psychopathological issues and deficits in emotion regulation.

## AUTHOR CONTRIBUTIONS

**Evelyn Mary‐Ann Antony**: Conceptualization; Data curation; Formal analysis; Investigation; Methodology; Project administration; Resources; Software; Validation; Visualization; Writing ‐ original draft; Writing ‐ review & editing. **Nadin Beckmann**: Methodology; Supervision; Writing ‐ review & editing. **Steve Higgins**: Methodology; Supervision; Writing ‐ review & editing.

## CONFLICT OF INTEREST STATEMENT

The authors have declared that they have no competing or potential conflicts of interest.

## Ethical considerations

Ethics was granted by the School of Education Research Ethics Committee, Durham University.

## Data Availability

Data sharing is not available for this article, as no data were created or analysed in this study.
